# Measles-Related Hospitalizations in Italy, 2004–2016: The Importance of High Vaccination Coverage

**DOI:** 10.5334/aogh.2455

**Published:** 2019-03-19

**Authors:** Fabiana Fiasca, Stefano Necozione, Leila Fabiani, Marianna Mastrodomenico, Antonella Mattei

**Affiliations:** 1 Department of Life, Health and Environmental Sciences, University of L’Aquila, IT

## Abstract

**Background:**

Measles is a highly contagious human infectious disease. It can lead to serious complications and often requires hospitalization. In Italy, as in other European countries, the goal of measles elimination in 2015 failed. To reach this target, identifying susceptible individuals, closing any immunity gaps and reaching adequate vaccination coverage is necessary.

**Objective:**

To contribute to these purposes, a retrospective observational study on measles-related hospitalization between 2004 and 2016 in Italy was conducted, using the national hospital discharge database as informational flow.

**Methods:**

Admission frequencies and hospitalization rates were compared between regions with low (<90%) and high (≥90%) vaccination coverage for measles at age 24 months. Categorical variables were analyzed using the χ2 test or the χ2 test for trend for ordinal variables; t test was performed to verify the significance when annual average hospitalization rates were compared. Trends of vaccination coverage and hospitalization rates were analyzed using the slope of the regression line.

**Findings:**

During the study period, 9,546 measles-related hospitalizations were collected in Italy, with an average annual number equal to 734. The overall measles hospitalization rates increased from 0.21 × 100,000 persons in 2004 to 0.82 × 100,000 in 2016 (β = 0.04; p = 0.689). A shift of mean age (from 1–17 years to 18–44 years) of measles-related hospitalizations was shown. A number of admissions for the low vaccination coverage group about twice as much as recorded for the other regional group was shown (6,344 vs 3,202). The involvement of 18–44 age class in the high vaccination coverage group was 14% higher compared with the low vaccination coverage group, in which, however, the 1–17 age class presented a 16% higher frequency.

**Conclusions:**

These findings confirmed that large measles epidemics continue to occur in Italy, although with regional differences related to different rates of measles immunization. Vaccination coverage >90% led to a halving of measles hospitalizations, but it is insufficient for the elimination: ≥95% coverage continues to be the target to be reached.

## Introduction

Measles is a highly contagious infectious disease, with an estimated transmissibility to susceptible contacts of 70–100% [1]. Due to this high transmissibility, a level of herd immunity equal to 96–98% is estimated to be necessary to protect from measles outbreaks [1]. Measles can lead to serious complications such as pneumonia, otitis media, keratoconjunctivitis, and encephalitis [2]. It still represents a serious public health problem worldwide, being responsible for more than 100,000 deaths every year [3].

In industrialized countries, between 1.4% and 19.0% of measles cases require hospitalization [4,5,6,7].

However, measles is a vaccine preventable disease and the incidence of infection declined markedly worldwide after the introduction of a live-attenuated vaccine [2].

At present, vaccination for measles is mandatory in Italy [8]. The Decree-Law 73/2017 increased the number of mandatory vaccinations from four to ten, for minors up to 16 years old. Vaccination against pertussis, measles, mumps, rubella (MMR), varicella, and *Haemophilus influenzae* type b (Hib) was added to the list of already mandatory vaccinations (diphtheria, tetanus, hepatitis B and polio) [8]. According to the law, all people who refused to be vaccinated could be subject to a fine, or children could be denied attendance to education services until the age of 6 years old [8].

The current Italian national vaccination schedule includes two doses of combined measles-mumps-rubella vaccine: the first dose administered at 12–15 months of age and the second dose at 5–6 years of age [9].

The Italian health system is highly decentralized. Decentralization means great variability of health services across regions [10]. Regional differences in policies and financing cause a large vertical fragmentation in the extent and the quality of health strategies between regions or local health authorities of excellence, which are mainly found in the northern part of the country, and the remaining area of the nation [10]. These differences in the health system between regions can also influence the different adherence to the vaccine supply in the different areas of the country.

The World Health Organization Regional Office for Europe set a target to eliminate measles by the end of the year 2015 but failed to reach this goal [11]. Italy is one of the European countries where measles is still an endemic infection [11].

In order to eliminate measles, it is necessary to identify susceptible individuals, close any immunity gaps and reach adequate vaccination coverage [2]. For this purpose, an epidemiological survey of measles-related hospitalizations in Italy was carried out.

## Materials and Methods

A retrospective observational study investigating hospitalizations for measles from 1 January 2004 to 31 December 2016 in Italy was performed.

The national hospital discharge database, held by the Ministry of Health (Ministry of Health – General Management of Health Planning – Hospital Discharge Records Database), was consulted to select the cases, coded according to the International Classification of Disease, Ninth Revision, Clinical Modification (ICD-9-CM) system. The hospital discharge registry contains information about each patient discharged from public and private hospitals; it includes data related to both clinical and organizational aspects of hospitalization, but it lacks information on laboratory-confirmed cases and vaccination status [12].

Measles-related codes include the following: post-measles encephalitis (055.0), post-measles pneumonia (055.1), post-measles otitis media (055.2), measles keratoconjunctivitis (055.71), measles with other specified complication (055.79), measles with unspecified complication (055.8) and measles without mention of complication (055.9). In this study, we included all admissions with at least one measles-related main or secondary discharge diagnosis.

Gender and age classes (<1 year, 1–17 years, 18–44 years, 45–64 years, 65–85 years, >85 years) of study population were analyzed. Data provided by the Ministry of Health did not contain any patient identifiers and was therefore completely anonymous. Ethical approval was therefore not required for this study.

Hospitalization rates were expressed × 100,000 persons. Population data for 2004–2016 was obtained from the Italian Institute of Statistics (ISTAT), which registers the national and regional population, by age group, as of the 1st of January for each year [13]. Surveillance data about vaccination coverage for measles at age 24 months was provided by the Italian Ministry of Health database [14]. The calculation of the coverage takes place on the basis of data on the vaccination activities that are sent to the Ministry of Health from autonomous regions and provinces, using a specific survey model [14]. The Ministry of Health publishes data on vaccination coverage for 24-month-old children, 36-month-old children (since 2016), 5–6-year-old children (since 2016), and 16–18-year-old adolescents (since 2017) [14].

Trends of vaccination coverage and hospitalization rates were analyzed using the slope of the regression line.

**Figure 1:**
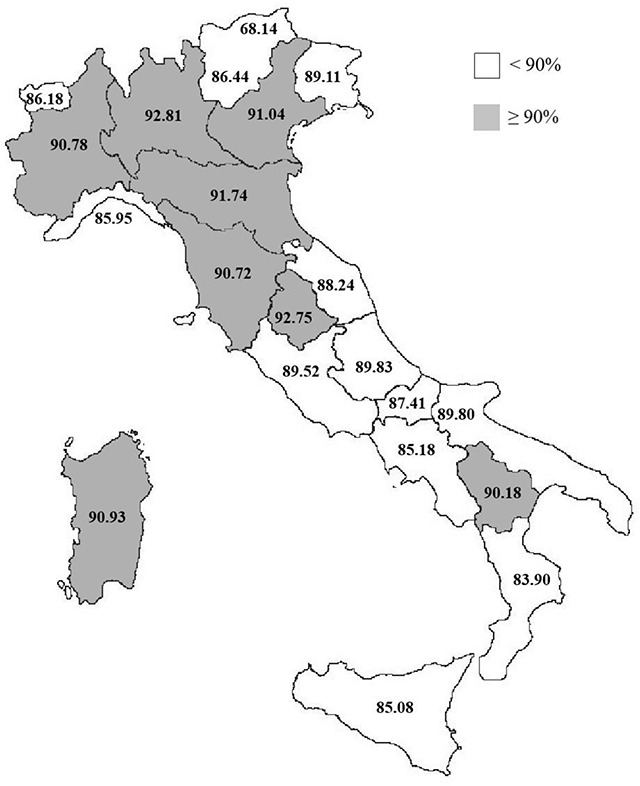
Stratification of Italian regions by vaccination coverage for measles at 24 months of age, calculated by averaging the coverage over the 13-year period 2004–2016. <90%: “low vaccination coverage” group; ≥90%: “high vaccination coverage” group.

Choosing a cut-off equal to 90% for the average vaccination coverage for measles during the study period, the Italian regions was stratified into two groups: those with an average vaccination coverage <90% (“low vaccination coverage” group) and those with an average vaccination coverage ≥90% (“high vaccination coverage” group) (Figure 1). Once the homogeneity of the distribution by sex and age of the reference population of these two regional groups was verified (Table 1), admission frequencies and average annual hospitalization rates between them were compared. Categorical variables were analyzed using the χ2 test or the χ2 test for trend for ordinal variables; t test was performed to verify the significance when average hospitalization rates were compared. All significant tests were two-sided. A value of p-value < 0.05 was considered significant. The statistical analyses were performed using STATA software package.

**Table 1:** Distribution by sex and age classes of reference population among low and high vaccination coverage groups (2004–2016).

	Average Annual Reference Population (2004-2016)	
	Low Vaccination Coverage Group N = 29,635,096	High Vaccination Coverage Group N = 30,330,148

**Gender, n (%)**		
Male	14,343,551 (48.40)	14,746,118 (48.62)
Female	15,291,545 (51.60)	15,584,030 (51.38)

**Age classes, n (%)**		
<1 year	270,165 (0.91)	270,033 (0.89)
1–17 years	5,015,831 (16.93)	4,585,337 (15.12)
18–44 years	10,821,250 (36.51)	10,682,995 (35.22)
45–64 years	7,762,485 (26.19)	8,276,815 (27.29)
65–85 years	5,171,505 (17.45)	5,809,795 (19.16)
>85 years	593,860 (2.00)	705,173 (2.32)

## Results

During the study period, 9,546 measles-related hospitalizations were collected in Italy, with an average annual number equal to 734 (Table 2). The 52.18% (4,981/9,546) of admissions involved male patients and the majority of hospitalizations (56.12%, 5,357/9,546) occurred in the 18–44-year age group. A total of 19 measles-related deaths were detected, but none of these in infants aged <1 year (data not shown).

**Table 2:** Distribution by sex and age classes of hospitalized measles cases among low and high vaccination coverage groups (2004–2016).

	TOTAL N = 9,546	Low Vaccination Coverage Group n = 6,344 (66.46%)	High Vaccination Coverage Group n = 3,202 (33.54%)	p-value

**Gender, n (%)**				0.042*

Male	4,981 (52.18)	3,357 (52.92)	1,624 (50.72)	
Female	4,565 (47.82)	2,987 (47.08)	1,578 (49.28)	

**Age classes, n (%)**				
<1 year	590 (6.18)	423 (6.67)	167 (5.22)	0.005*
1–17 years	3,082 (32.29)	2,402 (37.86)	680 (21.24)	<0.001*
18–44 years	5,357 (56.12)	3,271 (51.56)	2,086 (65.15)	<0.001*
45–64 years	364 (3.81)	181 (2.85)	183 (5.72)	<0.001*
65–85 years	129 (1.35)	55 (0.87)	74 (2.31)	<0.001*
>85 years	24 (0.25)	12 (0.19)	12 (0.37)	0.127*

* χ2 test or the χ2 test for trend.

**Figure 2:**
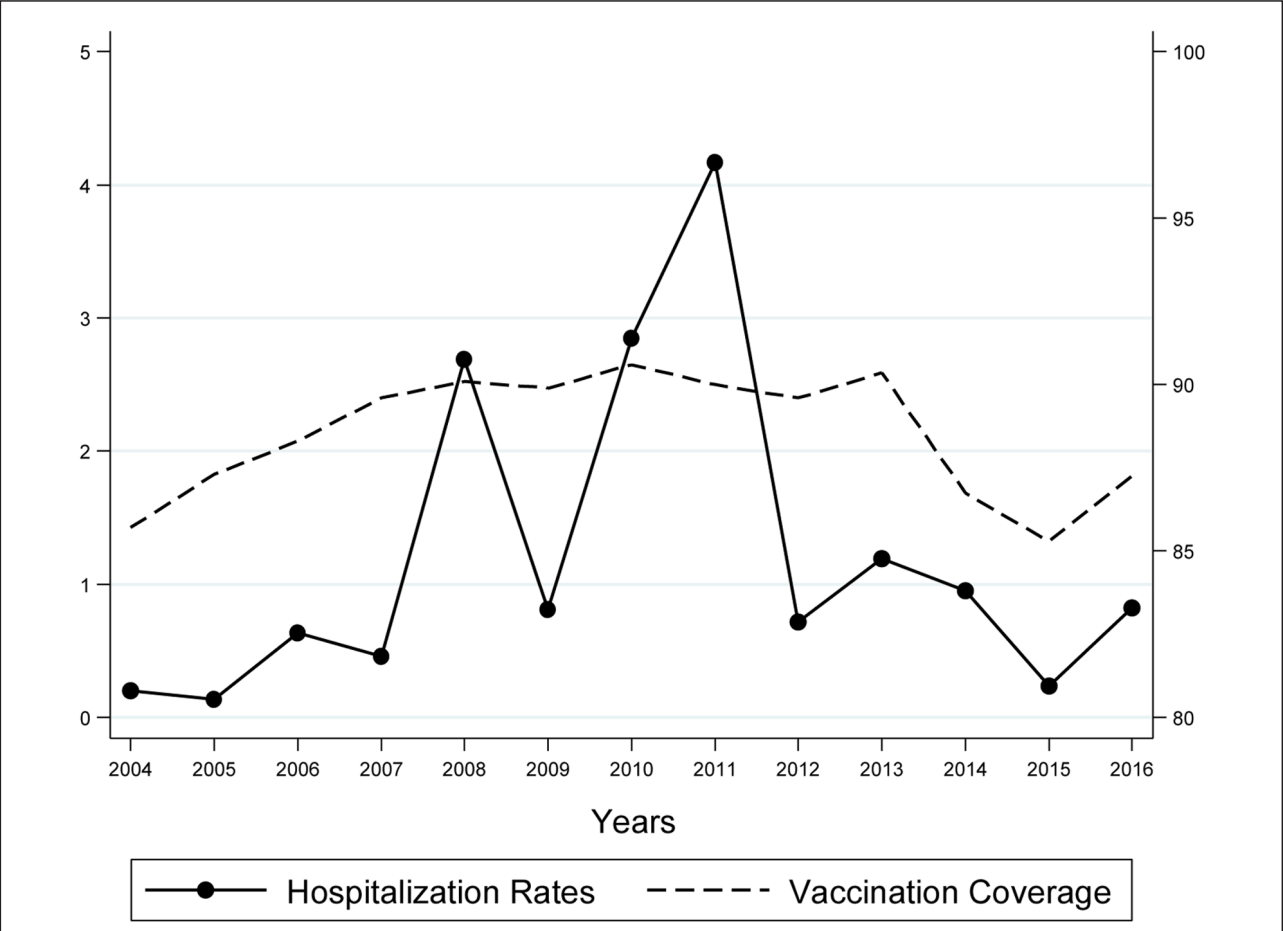
Temporal trend of hospitalization rates and vaccination coverage for measles (2004–2016). Trend test. Hospitalization rates: β coefficient = 0.04; p = 0.689. Vaccination coverage: β coefficient = –0.03; p = 0.834.

As shown in Figure 2, the overall measles hospitalization rates increased, over the study period, from 0.21 × 100,000 persons in 2004 to 0.82 × 100,000 in 2016 (β coefficient = 0.04; p = 0.689). The average hospitalization rate for the study timeframe amounted to 1.22 × 100,000 persons (Table 3), with fluctuations due to periodic measles outbreaks occurred in 2006, 2008, 2011 and 2013, with a maximum peak equal to 4.17 × 100,000 persons in 2011. The average vaccination coverage of 24-month-old children with the first dose of measles vaccine was 88.5%, with a slightly decreasing trend (β coefficient = –0.03; p = 0.834). No statistical significant trends emerged.

**Table 3:** Average annual hospitalization rates by sex and age classes among low and high vaccination coverage group (2004–2016).

Average Annual Hospitalization Rates × 100,000 persons				
	Italy	Low Vaccination Coverage Group	High Vaccination Coverage Group	p-value*

**Total**	1.22	1.34	0.77	**0.029**

**Gender**				
Male	1.31	1.80	0.85	**0.025**
Female	1.14	1.50	0.78	**0.036**

**Age classes**				
<1 year	8.35	12.05	4.76	**0.047**
1–17 years	2.47	3.68	1.14	**0.006**
18–44 years	1.92	2.33	1.50	0.135
45–64 years	0.17	0.18	0.17	0.706
65–85 years	0.09	0.08	0.10	0.288
>85 years	0.14	0.16	0.13	0.874

* t test.

**Figure 3:**
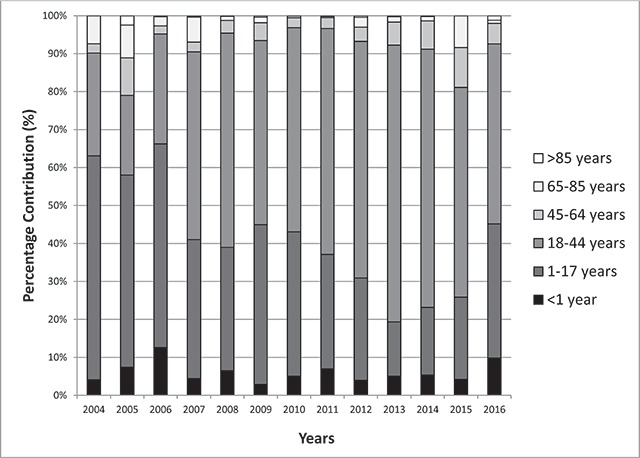
Percentage contribution to hospitalized cases of measles per year by age classes (2004–2016).

The percentage contribution per year by age groups was analyzed (Figure 3): a shift of mean age (from 1–17 years to 18–44 years) of measles-related hospitalizations was shown, particularly during measles outbreaks.

Through a comparison of measles-related hospitalizations between low and high vaccination coverage groups (Tables 2 and 3), a number of admissions for the low vaccination coverage group about twice as much as recorded for the other regional group was shown (6,344 vs. 3,202). A statistically significant difference (p = 0.042) according to gender emerged, with a male population more represented in the high vaccination coverage group (49.28% vs. 47.08%). Sixty-five percent of the admissions for this regional group affected the 18–44 age class versus a frequency of 51.56% recorded for the low vaccination coverage group (p < 0.001). The age class 1–17 years, furthermore, was more represented in the low vaccination coverage group than in the other one (37.86% vs. 21.24%, p < 0.001).

Children aged <1 year presented the highest average annual hospitalization rate (8.35 × 100,000 persons), followed by 1–17 age class (2.47 × 100,000 persons). In the low vaccination coverage group the value of the average annual hospitalization rate for infants in the first year of life was about three times higher than that of the other regional group (12.05 vs. 4.76, p = 0.047). The same ratio was found for the 1–17 age class (3.68 vs. 1.14, p = 0.006).

## Discussion

This study, to our knowledge, is the first that compare regions with low or high vaccination coverage for measles, analyzing measles-related hospitalizations in Italy over a period of 13 years, using national discharge data. Our findings confirmed that large measles epidemics continue to occur in Italy, although with regional differences related to different rates of measles immunization.

Our results showed that the hospitalization frequency significantly increased in adults aged 18–44 years over the study period, confirming an increase in susceptibility to measles that, in turn, reflected an epidemiological transition of mean age of infection towards older age groups, as expected when suboptimal vaccination coverage is maintained for a long period [15,16]. These evidence are similar to those emerged by Italian study on laboratory confirmed measles cases: a case-based surveillance of measles in Sicily showed that about half of hospitalized cases were among adults aged 19 years and older, whereas a study on the evaluation of measles and rubella integrated surveillance system in Apulia region demonstrated that the age class 15–39 years was the most affected age group [17,18].

This increase in susceptibility could be observed due to the fact that some adults have never been exposed to measles or have never been vaccinated; other possible reasons could be primary or secondary vaccine failures: they were vaccinated but did not respond or they suffered a decline of vaccine-induced immunity [15,16,17,18,19]. It was demonstrated that having receive the last dose of measles vaccine more than 10 years previously was associated to a small but significantly increased risk of becoming infected compared with a more recent administration of the vaccine [20].

On the other hand, a decreasing trend of coverage vaccination was shown: this could be partly related to growing concerns about vaccine safety, encouraged by anti-vaccination movements [15]. In Italy, the use of internet as a source of information about vaccines and a large number of children in a family emerged as the factors more associated with a missed vaccination [21].

Children aged <1 year presented the highest hospitalization rates, in line with the findings of other Western countries [22,23]. There are various reasons for this evidence. First of all, the actual vaccination policy that provides the administration of the first measles vaccine at the age of 13 months; secondly, contacts with adults susceptible to measles, which may represent an important reservoir for transmission; finally, the decline of indirect protection of infants through maternal antibodies transferred across the placenta and through the lactation [9,15,24]. In fact, measles-specific maternal antibodies decline gradually during the first year of life with the development of the infants’ own immune system, but they could neutralize the vaccine antigens before the development of a specific immune response if the measles vaccine is administrated at an early age [24]. Furthermore, the proportion of antibodies received from a mother with vaccine-induced immunity is lower than that transmitted from a naturally immunized mother and, in recent times, the mother is more likely to have acquired measles immunity through vaccination [25]. So the possible increasing gap of susceptibility due to the early loss of maternal antibodies is of increasing concern [24]. In addition, several studies reported a considerable delay in administration of the first dose of vaccine in infants, as emerged for the administration of other vaccines, which could increase this gap even more [26,27,28].

Consequently, the timing of vaccination should be carefully determined, and the interval between vaccination and the loss of maternally derived antibodies should be minimized to protect children from infection with measles.

In our study, the average hospitalization rate for infants aged <1 year in the low vaccination coverage group of regions was nearly one order of magnitude higher than that of the other group (12.05 vs. 4.76): this might suggest the need of the administration of a supplementary dose of measles vaccine from six months of age in this area, following the recommendations of the World Health Organization that provided the possibility of a supplementary dose from six months of age and before one year of age “during campaigns where the risk of measles among infants <9 months of age is high”, “for individual infants at high risk of contracting measles”, along with the improvement of the immunization activities [29]. However, the number of hospitalizations for the high vaccination coverage group was around half of that of the other group; this could positively impact the risk to contract nosocomial infection and this is especially important for immune-deficient patients, who cluster in hospitals [30]. Furthermore, several studies indicated the hospital exposure as one of the main cause of measles outbreaks: reducing hospitalizations could contribute to limit measles transmission [31].

In Italy, hospitalizations have been estimated to account for 40–50% of direct costs of measles cases [30,31,32]. So a limited number of hospitalizations, as shown for the high vaccination coverage group of regions, implies a reduction of health costs for measles.

An average hospitalization rate equal to 1.22 × 100,000 persons was found. Considering that globally, measles incidence was usually expressed as cases per million population, this result suggested high rates of transmission in the community [15]. Additionally, an average hospitalization rate equal to 0.77 × 100,000 persons in the high vaccination coverage group indicated that a vaccination coverage ≥90% but less than 95% was insufficient for elimination. However, it is necessary to reach and maintain levels of immunity ≥95% in each birth cohort to avoid new reservoirs of susceptible children [7,33]. Indeed, it was documented that ≥95% of a population is needed to be immune in order to achieve herd immunity: this means stopping measles endemic transmission and protecting those subjects who cannot be vaccinated [1,30,34].

Two important figures emerged from this investigation: the involvement of the 18–44 age class in the high vaccination coverage group was 14% higher compared with the low vaccination coverage group, in which, however, 1–17 age class presented a frequency 16% greater.

With regard to the first point, taking into consideration the high susceptibility to measles in the 18–44 age group, strategy to catch up these subjects should be enforced. It was proposed to use the occasion of the pap-test screening or visits performed during sport activities to promote the vaccination [35].

The greater involvement of the 1–17 age class in the low vaccination coverage group implies the need to improve immunization activities in this area to reduce the risk of contracting measles in the population targeted by vaccination campaigns. To ensure this, it should be considered that the parents’ worldwide acceptance of childhood immunization is a key issue. The lack of knowledge of the seriousness of the disease, skepticism about the vaccination benefits, and increased fear of adverse events following immunization are the main reasons of the decreased adherence to measles vaccination [33].

Consequently, in order to implement vaccination coverage, providing accurate information about clinical course of the disease and vaccine safety are crucial topics for healthcare professionals.

*Limitations of the study.* Using the hospital discharge database as informational flow, some diagnoses could be excluded or misattributed [36]. Nevertheless, as can be deduced from the consultation of the annual reports on hospitalization published by the Ministry of Health, the Italian hospital discharge database between 2004 and 2016 was characterized by a high level of completeness, with an average of 97.4% considering public and accredited private institutions, and an average error rate of 4.6% over the period considered, and with a minimum value of 0.6% achieved in 2016 [37]. The quality level of the hospital discharge database, therefore, is extremely high.

Furthermore, since the study was on national dimension, it is unlikely that this possible bias changed our final results [38]. However, as these findings were based on admission data, we took only severely affected subjects into account, excluding cases of mild measles that did not require hospital care. Additionally, the vaccination status of each case included in the analysis was not consider, as these data were not available. Finally, hospital discharge records did not allow to discriminate between nosocomial transmission of the infection and community acquired infection [38].

## Conclusions

In Italy, measles still represents a serious public health problem. For this purpose, countries aiming to eliminate measles, as Italy, should activate and implement catch-up, keep-up, and follow-up immunization campaigns, in order to reach and maintain a vaccination coverage ≥95% with the measles vaccine [19].

## References

[1] Voigt EA, Ovsyannikova IG, Haralambieva IH, et al. Vaccine. 2016; 34(41): 4913–4919. DOI: 10.1016/j.vaccine.2016.08.060 27591105 PMC5278779

[2] Lancella L, Di Camillo C, Vittucci AC, Boccuzzi E, Bozzola E and Villani A. Measles lessons in an anti-vaccination era: Public health is a social duty, not a political option. Ital J Pediatr. 2017; 43(1): 102 DOI: 10.1186/s13052-017-0420-6 29141656 PMC5688720

[3] Moss WJ. Measles. Lancet. 2017; 390(10111): 2490–2502. DOI: 10.1016/S0140-6736(17)31463-0 28673424

[4] Godoy P, Domínguez A, Alvarez J, et al. Measles epidemiology in Catalonia (Spain): Implications for a regional vaccination programme. Int J Epidemiol. 1999; 28(3): 558–562. DOI: 10.1093/ije/28.3.558 10405864

[5] Lee B, Ying M, Papania MJ, Stevenson J, Seward JF and Hutchins SS. Measles hospitalizations, United States, 1985–2002. J Infect Dis. 2004; 189(suppl 1): S210–S215. DOI: 10.1086/381555 15106113

[6] Beutels P and Gay NJ. Economic evaluation of options for measles vaccination strategy in a hypothetical Western European country. Epidemiol Infect. 2003; 130(2): 273–283. DOI: 10.1017/S0950268802008142 12729196 PMC2869963

[7] Antona D, Lévy-Bruhl D, Baudon C, et al. Measles elimination efforts and 2008–2011 outbreak, France. Emerg Infect Dis. 2013; 19(3): 357–364. DOI: 10.3201/eid1903.121360 23618523 PMC3647670

[8] Ministry of Health. Decree Law 7 June 2017, n. 73, Urgent provisions on vaccination prevention, as amended by the conversion law July 31, 2017. http://www.trovanorme.salute.gov.it/norme/dettaglioAtto?id=60201. Accessed October 15, 2018.

[9] Ministry of Health. Vaccination Schedule of the National Vaccine Prevention Plan 2017–2019. http://www.salute.gov.it/imgs/C_17_pagineAree_4829_listaFile_itemName_0_file.pdf. Accessed October 15, 2018.

[10] Cicchetti A and Gasbarrini A. The healthcare service in Italy: Regional variability. Eur Rev Med Pharmacol Sci. 2016; 20(1 Suppl): 1–3. https://www.europeanreview.org/wp/wp-content/uploads/The-healthcare-service-in-Italy-regional-variability.pdf. Accessed November 13, 2018. 28083867

[11] World Health Organization Regional Office for Europe (WHO/Europe). 4th Meeting of the European Regional Verification Commission for Measles and Rubella Elimination (RVC). Copenhagen: WHO/Europe; 2015 http://www.euro.who.int/__data/assets/pdf_file/0011/304958/4th-RVC-meeting-report.pdf. Accessed September 17, 2018.

[12] Ministry of Health. Hospital Discharge Record. http://www.salute.gov.it/portale/temi/p2_6.jsp?lingua=italiano&id=1232&area=ricoveriOspedalieri&menu=vuoto. Accessed February 8, 2019.

[13] Italian Institute of Statistics. http://demo.istat.it/. Accessed October 1, 2018.

[14] Italian Ministry of Health. Childhood vaccination. http://www.salute.gov.it/portale/documentazione/p6_2_8_3_1.jsp?lingua=italiano&id=20. Accessed September 30, 2018.

[15] Berti E, Sollai S, Orlandini E, Galli L, DE Martino M and Chiappini E. Analysis of measles-related hospitalizations in Tuscany from 2000 to 2014. Epidemiol Infect. 2016; 144(12): 2605–12. DOI: 10.1017/S0950268816001023 27240964 PMC9150479

[16] Bechini A, Levi M, Boccalini S, et al. Progress in the elimination of measles and congenital rubella in Central Italy. Hum Vaccin Immunother. 2013; 9(3): 649–56. DOI: 10.4161/hv.23261 23292174 PMC3891724

[17] Tramuto F, Maida CM, Pojero F, et al. Case-based surveillance of measles in Sicily during 2012–2017: The changing molecular epidemiology and implications for vaccine strategies. PLoS One. 2018; 13(4): e0195256 DOI: 10.1371/journal.pone.0195256 29617454 PMC5884552

[18] Turiac IA, Fortunato F, Cappelli MG, et al. Evaluation of measles and rubella integrated surveillance system in Apulia region, Italy, 3 years after its introduction. Epidemiol Infect. 2018; 146(5): 594–599. DOI: 10.1017/S0950268818000407 29532766 PMC9134572

[19] Orenstein WA, Strebel PM, Papania M, Sutter RW, Bellini WJ and Cochi SL. Measles eradication: Is it in our future? Am J Public Health. 2000; 90: 1521–5. DOI: 10.2105/AJPH.90.10.1521 11029981 PMC1446359

[20] European Centre for Disease Control and Prevention. Surveillance report European monthly measles monitoring (EMMO). Stockholm 2012; 9: 1–10. http://ecdc.europa.eu/en/publications/Publications/SUR-EMMO-European-monthlymeasles-monitoring-March-2012.pdf. Accessed November 12, 2018.

[21] Restivo V, Napoli G, Marsala MG, et al. Factors associated with poor adherence to MMR vaccination in parents who follow vaccination schedule. Hum Vaccin Immunother. 2015; 11: 140–145. DOI: 10.4161/hv.34416 25483527 PMC4514278

[22] European Centre for Disease Prevention and Control. Measles surveillance data. http://ecdc.europa.eu/en/healthtopics/measles/epidemiological_data/pages/annual_epidemiological_reports.aspx. Accessed November 3, 2018.

[23] Chiew M, et al. Australian vaccine preventable disease epidemiological review series: Measles 2000–2011. Commun Dis Intell Q Rep. 2015; 39(1): E1–9. http://www.health.gov.au/internet/main/publishing.nsf/Content/cda-cdi3901-pdf-cnt.htm/$FILE/cdi3901a.pdf. Accessed October 4, 2018. 26063085 10.33321/cdi.2015.39.1

[24] Leuridan E, Hens N, Hutse V, Ieven M, Aerts M and Van Damme P. Early waning of maternal measles antibodies in era of measles elimination: Longitudinal study. BMJ. 2010; 340: c1626 DOI: 10.1136/bmj.c1626 20483946

[25] Eom H, Park Y, Kim J, et al. Occurrence of measles in a country with elimination status: Amplifying measles infection in hospitalized children due to imported virus. PLoS One. 2018; 13(2): e0188957 DOI: 10.1371/journal.pone.0188957 29447169 PMC5813900

[26] Akmatov MK, Kretzschmar M, Kramer A and Mikolajczyk RT. Timeliness of vaccination and its effects on fraction of vaccinated population. Vaccine. 2008; 26(31): 3805–11. DOI: 10.1016/j.vaccine.2008.05.031 18565626

[27] Luman ET and Chu SY. When and why children fall behind with vaccinations: Missed visits and missed opportunities at milestone ages. Am J Prev Med. 2009; 36(2): 105–11. DOI: 10.1016/j.amepre.2008.09.035 19062241

[28] Giuliani AR, Mattei A, Appetiti A, et al. Spontanuous demand For meningococcal B vaccination: Effects on appropriateness and timing. Hum Vaccin Immunother. 2018; 14(8): 2075–2081. DOI: 10.1080/21645515.2018.1466015 29927693 PMC6150011

[29] World Health Organization. Measles vaccines: WHO position paper – April 2017. Wkly Epidemiol Rec. 2017 4 28; 92(17): 205–27. https://apps.who.int/iris/bitstream/handle/10665/255149/WER9217.pdf;jsessionid=81273BFF7B4DA066730E4B5C0CC04E3E?sequence=1. Accessed February 6, 2019.28459148

[30] Celesia BM, Fontana R, Pinzone MR, et al. A measles outbreak in Catania, Sicily: The importance of high vaccination coverage and early notification of cases for health and economic reasons. Infez Med. 2014 9; 22(3): 222–6. 25269964

[31] Zhang RQ, Li HB, Li FY, Han LX and Xiong YM. Epidemiological characteristics of measles from 2000 to 2014: Results of a measles catch-up vaccination campaign in Xianyang, China. J Infect Public Health. 2017; 10(5): 624–629. DOI: 10.1016/j.jiph.2017.02.005 28254459

[32] Filia A, Brenna A, Panà A, Cavallaro GM, Massari M and Ciofi degli Atti ML. Health burden and economic impact of measles-related hospitalizations in Italy in 2002–2003. BMC Public Health. 2007; 7: 169 DOI: 10.1186/1471-2458-7-169 17650298 PMC1963450

[33] Bozzola E, Bozzola M, Calcaterra V, Barberi S and Villani A. Infectious diseases and vaccination strategies: How to protect the “unprotectable”? ISRN Prev Med. 2013; 2013. DOI: 10.5402/2013/765354 PMC4062883 24977097

[34] Magurano F, Baggieri M, Filia A, et al. Towards measles elimination in Italy: Virological surveillance and genotypes trend (2013–2015). Virus Res. 2017; 236: 24–29. DOI: 10.1016/j.virusres.2017.05.009 28522332

[35] Bechini A, Boccalini S, Tiscione E, et al. Progress towards measles and rubella elimination in Tuscany, Italy: The role of population seroepidemiological profile. Eur J Public Health. 2012; 22(1): 133–9. DOI: 10.1093/eurpub/ckq134 20880991

[36] Mattei A, Angelone AM, Michetti M, et al. Epidemiological impact of RV gastroenteritis in the Abruzzo Region: SDO analysis. Ann Ig. 2009; 21(1): 41–9. http://www.seu-roma.it/riviste/annali_igiene/apps/autos.php?id=584. Accessed November 21, 2018. 19385333

[37] Ministry of Health. Annual Reports. http://www.salute.gov.it/portale/temi/p2_6.jsp?lingua=italiano&id=1237&area=ricoveriOspedalieri&menu=vuoto. Accessed February 8, 2019.

[38] Fiasca F, Mattei A, Vittorini P, et al. Bacterial Meningitis Hospitalizations after the 2009 L’Aquila Earthquake: A Retrospective Observational Study. Asian J Epidemiol. 2018; 11(1): 46–51. DOI: 10.3923/aje.2018.46.51

